# Isolated Brachiocephalic Artery Dissection after Two-Debranching Thoracic Endovascular Aortic Repair: A Case Report

**DOI:** 10.3400/avd.cr.26-00032

**Published:** 2026-06-26

**Authors:** Reiko Kemmochi, Kosuke Ujihira, Noriyuki Tokunaga

**Affiliations:** Department of Cardiovascular Surgery, Tsuyama Chuo Hospital, Tsuyama, Okayama, Japan

**Keywords:** brachiocephalic artery dissection, thoracic endovascular aortic repair, endovascular repair

## Abstract

An 87-year-old man with a distal aortic arch aneurysm underwent 2-debranching thoracic endovascular aortic repair (TEVAR). Five days postoperatively, he developed sudden chest pain and syncope, with severe upper-limb hypoperfusion. Contrast-enhanced computed tomography (CECT) revealed brachiocephalic artery (BCA) dissection compressing the true lumen. Although conservative management was initially selected, percutaneous transluminal angioplasty with kissing bare-metal stents from the BCA to the right subclavian and right common carotid arteries was performed for recurrent hypotension. Postprocedural CECT confirmed satisfactory stent expansion and graft patency. This case demonstrates that careful monitoring enables timely intervention for BCA dissection after TEVAR.

## Introduction

Thoracic endovascular aortic repair (TEVAR) is widely adopted in the treatment of thoracic aortic diseases. Stent graft-induced new entry (SINE) is a well-established complication associated with dissection after TEVAR, and when it occurs proximally, it can progress to retrograde type A aortic dissection (RTAD).^1,2)^ Comparatively, isolated brachiocephalic artery (BCA) dissection, localized without aortic arch involvement, following TEVAR is rare but potentially under-recognized. Herein, we report the case of an 87-year-old man who developed isolated BCA dissection 5 days after 2-debranching TEVAR, and presented with upper extremity hypoperfusion and graft flow impairment.

## Case Report

**Fig. 1:**
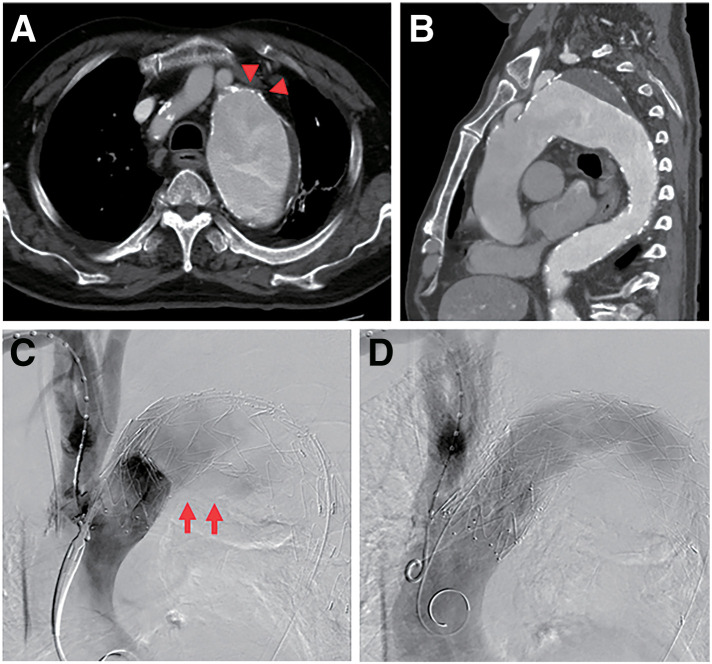
Preoperative CECT and intraoperative aortography during 2-debranching TEVAR. (**A**) Axial and (**B**) sagittal CECT showing a 67-mm distal aortic arch aneurysm with calcification in the proximal landing zone (arrowheads). (**C**, **D**) Intraoperative aortography during 2-debranching TEVAR. (**C**) Type Ia endoleak after initial proximal stent graft deployment (arrows). (**D**) Resolution of the endoleak after additional relining. No evidence of brachiocephalic artery dissection at this stage. CECT: contrast-enhanced computed tomography; TEVAR: thoracic endovascular aortic repair

An 87-year-old man with a medical history of hypertension and coronary vasospastic angina was admitted to our internal medicine department for the treatment of pneumonia. Contrast-enhanced computed tomography (CECT) revealed a fusiform aneurysm of the distal aortic arch with a maximum short diameter of 67 mm (**Fig. 1A** and **1B**). The proximal landing zone measured 21 mm in length from the origin of the BCA to the aneurysm, with reverse tapering and scattered calcifications. Given his advanced age, elective 2-debranching TEVAR was planned after recovery from pneumonia. Under general anesthesia, a right axillary artery–left common carotid artery (LCCA)–left axillary artery bypass was performed using an 8-mm T-shaped Propaten graft (W. L. Gore & Associates, Flagstaff, AZ, USA), and a pigtail catheter was inserted via the right radial artery (RA) for contrast injection. TEVAR was performed subsequently using 2 RelayPro NBS aortic endografts (28-N4-42-204-38S and 28-N4-42-204-42S; Terumo Aortic, Sunrise, FL, USA) under rapid pacing, with the proximal end extending slightly into the BCA. Proximal oversizing was 17.0% at the BCA origin (38 × 32 mm in diameter) and 13.3% (39 × 34 mm in diameter) at the central neck, balancing sealing requirements with the risk of retrograde dissection. Aortography revealed a type Ia endoleak (**Fig. 1C**), prompting subsequent deployment of a Zenith Alpha distal extension (ZTA-DE-42-94-W1; Cook Medical, Bloomington, IN, USA) for relining. The Zenith Alpha distal extension was selected to minimize the risk of further intimal injury, as it lacks proximal bare stents and anchors. Final angiography confirmed complete resolution of the endoleak with no signs of BCA dissection (**Fig. 1D**). The procedure was completed with coil embolization for the left subclavian artery and LCCA.

**Fig. 2:**
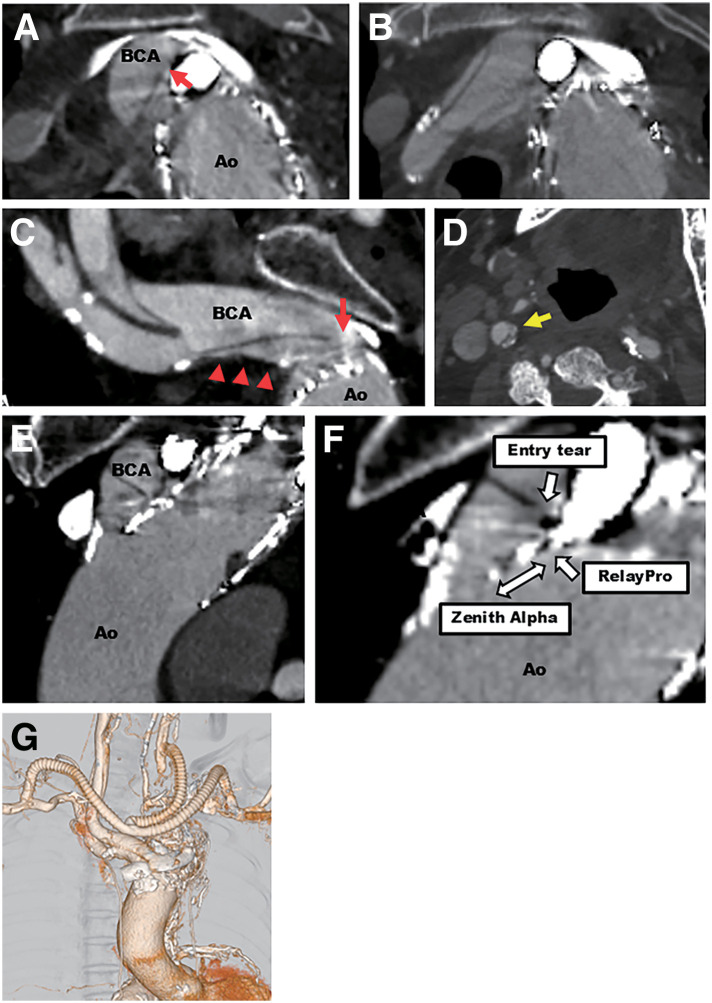
Contrast-enhanced computed tomography performed on postoperative day 5 at the onset of a transient loss of consciousness. (**A**, **B**) Axial and (**C**) coronal images showing dissection of the brachiocephalic artery with an entry tear near its origin (red arrow) and severe compression of the true lumen by the false lumen (red arrowheads). (**D**) Axial image at the level of the right internal carotid artery showing re-entry (yellow arrow) with preserved distal flow despite true lumen narrowing. (**E**) Oblique view confirming that the dissection is localized to the BCA. (**F**) Positional relationship between the stent grafts and the entry tear. (**G**) Volume-rendering image showing enhancement of the debranching bypass graft. Ao: aorta; BCA: brachiocephalic artery

Postoperatively, the patient recovered in the absence of any neurological deficits and was mobilized uneventfully. However, on postoperative day 5, he experienced sudden chest pain followed by a transient loss of consciousness. Bilateral upper extremity blood pressure was immeasurable, with weak RA pulses, although lower extremity pressures remained normal, thereby indicating severe upper body hypoperfusion. In response to rapid fluid administration, the patient’s consciousness was restored, with a systolic blood pressure of 40–50 mmHg in the upper extremities and 140 mmHg in the lower extremities. CECT revealed dissection of the BCA with an entry tear at its origin and compression of the true lumen by a false lumen (**Fig. 2A**–**2C**). Blood flow through the right common carotid artery (RCCA) was maintained via re-entry from the right internal carotid artery (**Fig. 2D**). There was no dissection to the ascending or arch aorta, and the entry tear was closer to the RelayPro proximal edge than to the Zenith Alpha (**Fig. 2E** and **2F**). Although the debranching bypass graft was enhanced on imaging (**Fig. 2G**), blood flow appeared compromised due to a true lumen narrowing.

**Fig. 3:**
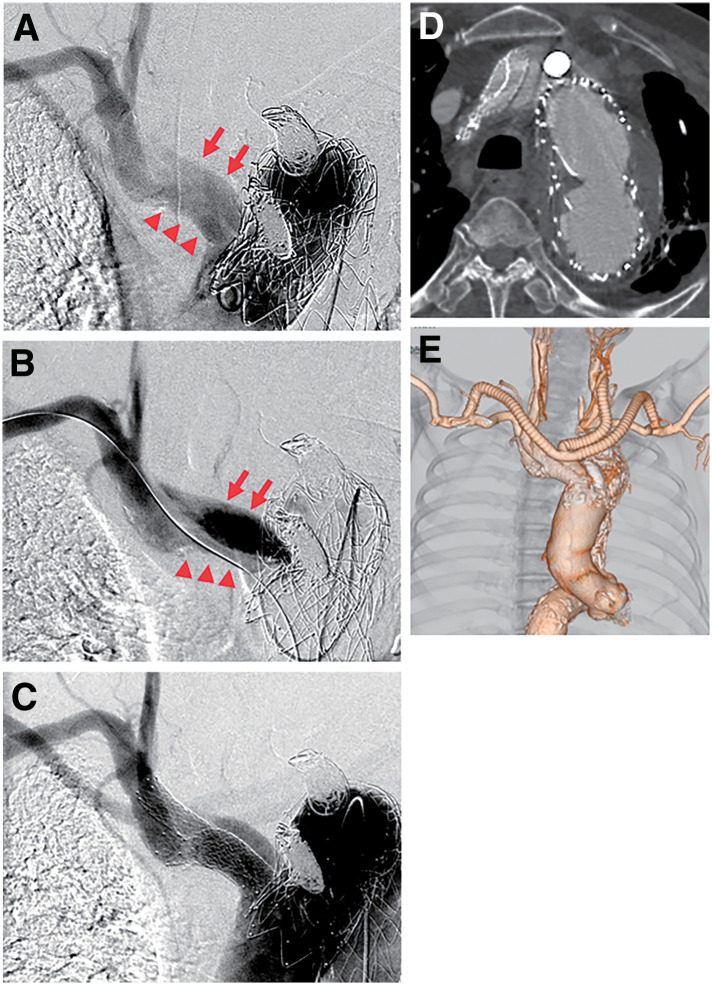
Angiographic findings during re-intervention and postoperative CECT. (**A**) Aortography on the day of symptom onset revealing BCA dissection with visualization of the false lumen (arrows) and preserved true lumen flow (arrowheads). (**B**) Selective angiography performed the following day showing a progression of false lumen expansion (arrows) and worsening true lumen compression (arrowheads). (**C**) Aortography after kissing stent placement from the BCA to the right subclavian and right common carotid arteries, revealing a restoration of true lumen flow without extension into the aorta. (**D**) Axial CECT prior to discharge showing adequate stent expansion. (**E**) Volume-rendering image confirming stent patency and bypass graft enhancement. CECT: contrast-enhanced computed tomography; BCA: brachiocephalic artery

Emergent aortography was performed. Upon arrival at the operating room, the patient’s upper-limb blood pressure had recovered. Under general anesthesia, aortography via the right common femoral artery (RCFA) confirmed preserved true lumen flow with delayed false lumen filling (**Fig. 3A**). Given that the pressure discrepancy had been resolved and entry closure was deemed difficult, the surgical team opted to undertake initial conservative management. After recovery from anesthesia, the fully conscious patient was admitted to the intensive care unit. Continuous arterial pressure monitoring was maintained via right RA and RCFA lines. Although neurological status remained stable, transient loss of the right RA pulse occurred twice overnight, resolving spontaneously. Given the recurrent upper-limb hypotension and the dependence of left cerebral circulation on graft flow, percutaneous transluminal angioplasty was performed on the following day.

A 9-Fr sheath was inserted via the right brachial artery and the left common femoral artery (LCFA) by cutdown. Selective angiography of the RCCA revealed worsening true lumen compression (**Fig. 3B**), and given that, owing to aortic arch curvature, cannulation to the BCA via the femoral approach was unsuccessful, the RCCA was surgically exposed at the level of the hyoid bone. A pull-through guidewire was placed from the LCFA to the RCCA via the true lumen, and an Epic vascular self-expanding stent system (12 × 60 mm; Boston Scientific, Marlborough, MA, USA) was deployed in a kissing manner in the BCA–right subclavian artery and BCA–RCCA segments, avoiding extension into the aorta (**Fig. 3C**). Post-deployment angiography and intravascular ultrasound confirmed adequate stent expansion.

Postprocedurally, the patient was mobilized in the absence of any further complications. Ankle-brachial index (ABI) measurements were 1.19 (right) and 1.10 (left), with no significant discrepancy between upper limbs. CECT scan 5 days after reintervention confirmed satisfactory stent expansion and graft patency (**Fig. 3D** and **3E**). There was no recurrence of the differential upper- and lower-limb pressures or any central nervous system disorders, and the patient recovered uneventfully. He was discharged 11 days after reintervention, and a follow-up CECT performed 3 months later confirmed continued stent expansion and graft patency.

## Discussion

We report a case of symptomatic isolated BCA dissection occurring days after 2-debranching TEVAR for a distal arch aneurysm. The incidence of fatal RTAD as a dissecting complication after TEVAR has been reported to be approximately 2%–5%.^2)^ However, there have been few reports of isolated BCA dissection.

In the patient described herein, a new entry was observed at the origin of the BCA, for which the potential underlying mechanisms included mechanical intimal injury associated with pigtail catheter manipulation or localized wall stress concentration caused by mechanical redistribution after TEVAR. Given that no guidewire misplacement or resistance to catheter manipulation occurred during the procedure, and that the onset was several days after surgery, the former is considered unlikely. The latter, localized wall stress concentration, could be attributable to factors similar to those observed in SINE and RTAD, including stent graft oversizing, radial force, and straightening force associated with the curvature of the aortic arch.^1,2)^ Furthermore, mechanical studies have shown that stress concentration tends to occur at vascular bifurcations.^3,4)^ In the present case, in addition to the oversizing rate of the stent graft at the BCA origin (17.0%), it is possible that the double layering of the RelayPro and Zenith Alpha stent grafts may have amplified radial and straightening forces. Retrospective review revealed that the proximal end of the RelayPro NBS was in close proximity to the entry tear at the posterior wall of the BCA origin, and that the BCA laceration occurred along the edge of the RelayPro NBS rather than the Zenith Alpha. This indicates that the primary trigger was a proximal SINE induced by the RelayPro edge, likely exacerbated by the increased radial and straightening forces following the addition of the Zenith Alpha. Moreover, calcification and stiffening of the proximal landing zone may have reduced the elasticity of the arterial wall, thereby increasing rigidity, reducing compliance, and influencing pressure propagation and hemodynamics.^5,6)^ Collectively, these factors may have contributed to an accumulation of stress at the origin of the BCA, leading to dissection. In the acute phase, although upper-limb hypoperfusion was observed, these symptoms temporarily resolved spontaneously. Emergency aortography confirmed that true blo od flow was maintained, and initial conservative management was selected, with stringent blood pressure control and intensive monitoring enabled timely reintervention when necessary.

Given the rarity of isolated BCA dissection, there are as yet no standard treatment guidelines. Although conservative management, including stringent blood pressure control, has contributed to favorable outcomes in asymptomatic cases,^7)^ progression to type A dissection has also been reported, and to date no consensus has been reached regarding the most appropriate therapeutic approach.^8)^ Neri et al. recommend intervention for carotid hypoperfusion, symptomatic subclavian steal syndrome, transient ischemic attack, and embolic symptoms.^9)^ In this patient, true lumen blood flow was preserved and neurological symptoms rapidly improved, so immediate intervention was not performed. Follow-up focused on careful evaluation of blood flow and neurological stability, with a plan for reintervention if necessary. Blood flow was ultimately stabilized with endovascular stenting.

While we considered attempting complete entry closure with covered stents or Zone 0 extensions (e.g., chimney or branched grafts), the former was deemed technically unreliable due to the dilated BCA (21 mm), and the latter carried a high risk due to the patient’s advanced age and mildly dilated ascending aorta (43 mm), which increased the potential for procedure-related RTAD. Furthermore, endovascular treatment was selected over open surgery due to its significantly lower invasiveness. Consequently, we prioritized a less-invasive endovascular approach using a bare-metal stent. Although a patent entry carries a potential risk of future aneurysmal expansion, our primary goal was the immediate restoration of cerebral and upper-limb perfusion. Based on these considerations, careful follow-up is essential to enable monitoring for false lumen expansion, aneurysmal development, in-stent restenosis, and delayed RTAD. Limitations of this report include its single-case nature, the unknown mid- to long-term postoperative course, and the fact that the cause was only indirectly inferred at the time of the initial TEVAR. Nevertheless, this case may provide valuable insights into the management of a rare post-TEVAR complication.

## Conclusion

Isolated BCA dissection after 2-debranching TEVAR is a particularly rare event. Although in cases in which true lumen flow is preserved, initial conservative management may serve as a reasonable treatment option, recurrent hypoperfusion or low graft-dependent cerebral circulation warrants prompt reintervention, and careful follow-up is essential.
